# Supplementary material for: Blueberry Anthocyanins from Commercial Products: Structure Identification and Potential for Diabetic Retinopathy Amelioration
Source: Molecules. 2022 Nov 2;27(21):7475. doi: 10.3390/molecules27217475 (PMC9655577; doi:10.3390/molecules27217475)
Supplement: Supplementary file 1 [file molecules-27-07475-s001.zip › molecules-1973259-Supplementary Material.pdf]

Supplementary Material

Blueberry Anthocyanins from Commercial Products: Structure Identification and Potential for Diabetic Retinopathy Amelioration

Rui Li [1,†], Zhan Ye [1,2,3,4,†], Wei Yang [1,2,3,4], Yong-Jiang Xu [1,2,3,4], Chin-Ping Tan [5] and Yuanfa Liu [1,2,3,4,*]

[1] School of Food Science and Technology, Jiangnan University, Wuxi 214122, China
[2] State Key Laboratory of Food Science and Technology, Jiangnan University, Wuxi 214122, China
[3] National Engineering Research Center for Functional Food, Jiangnan University, Wuxi 214122, China
[4] Collaborative Innovation Center of Food Safety and Quality Control in Jiangsu Province, Wuxi 214122, China
[5] Department of Food Technology, Faculty of Food Science and Technology, Universiti Putra Malaysia, Serdang 43400, Malaysia
* Correspondence: yfliu@jiangnan.edu.cn; Tel.: +86-0510-8587-6799
† These authors contributed equally to this work.

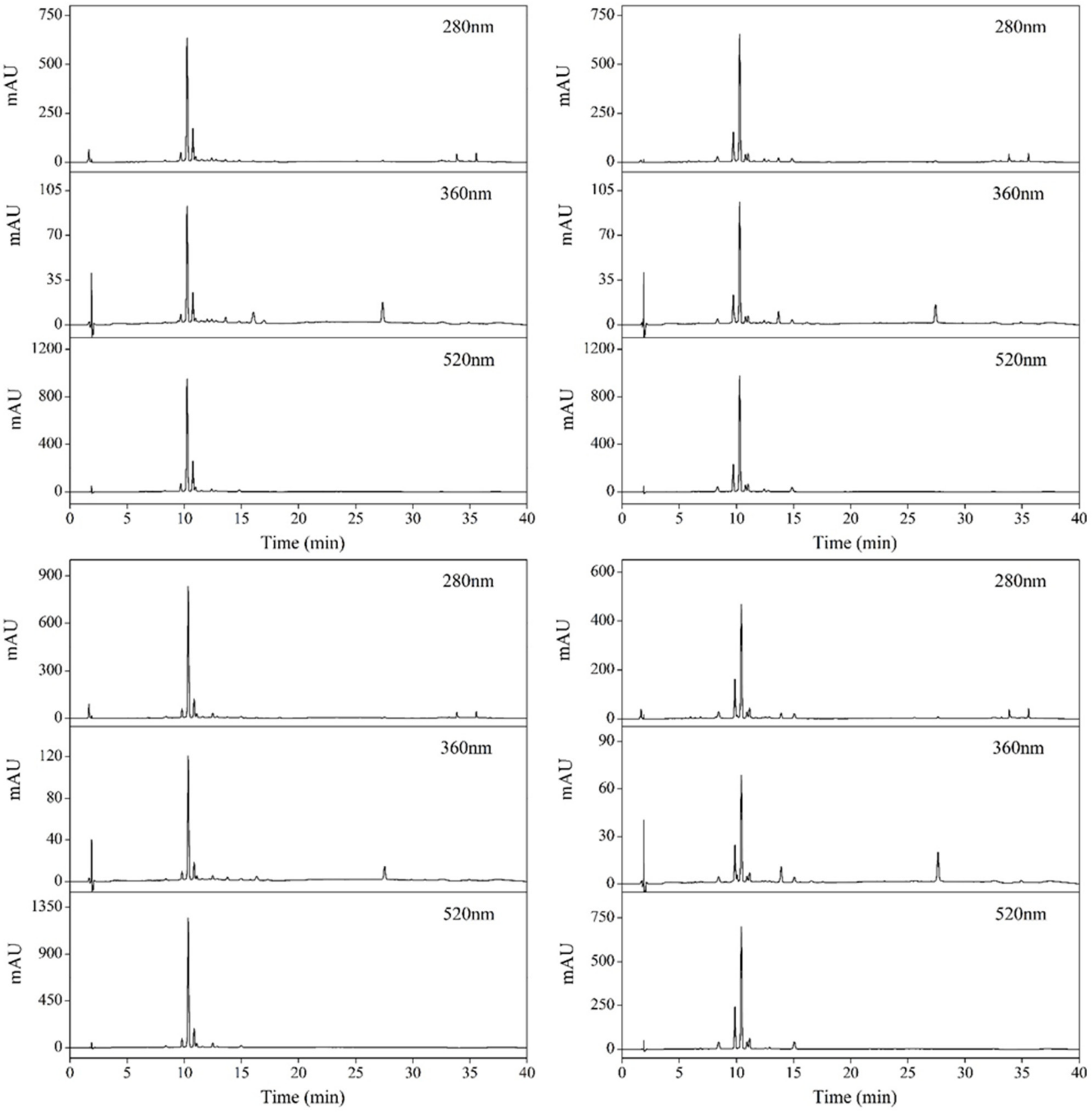

Figure S1. Chromatograms of extracts of blueberries monitored at 280 nm, 360 nm, and 520 nm for optimal detection of flavan-3-ols, flavonols and anthocyanins. S2-S5 were the chromatograms of the four different samples obtained by HPLC-DAD, respectively.

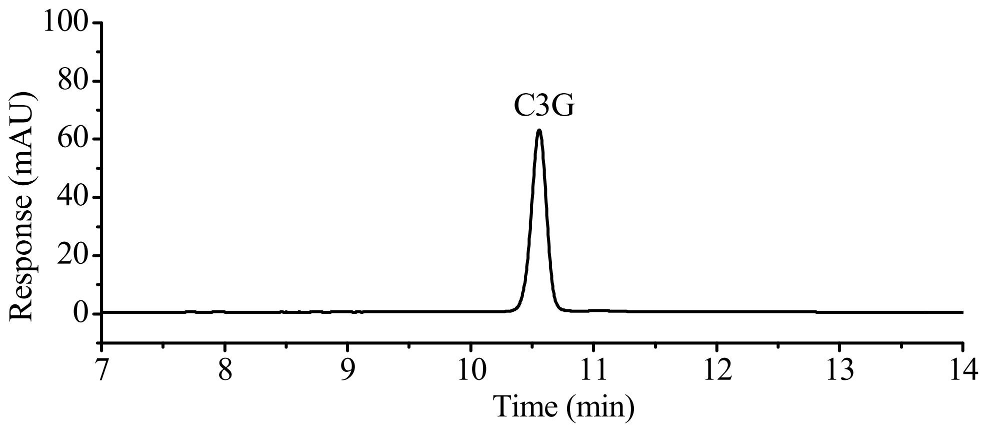

Figure S2. Chromatograms of extracts of Cyanidin-3-O-glucoside at 520 nm (RT = 10.55 min).

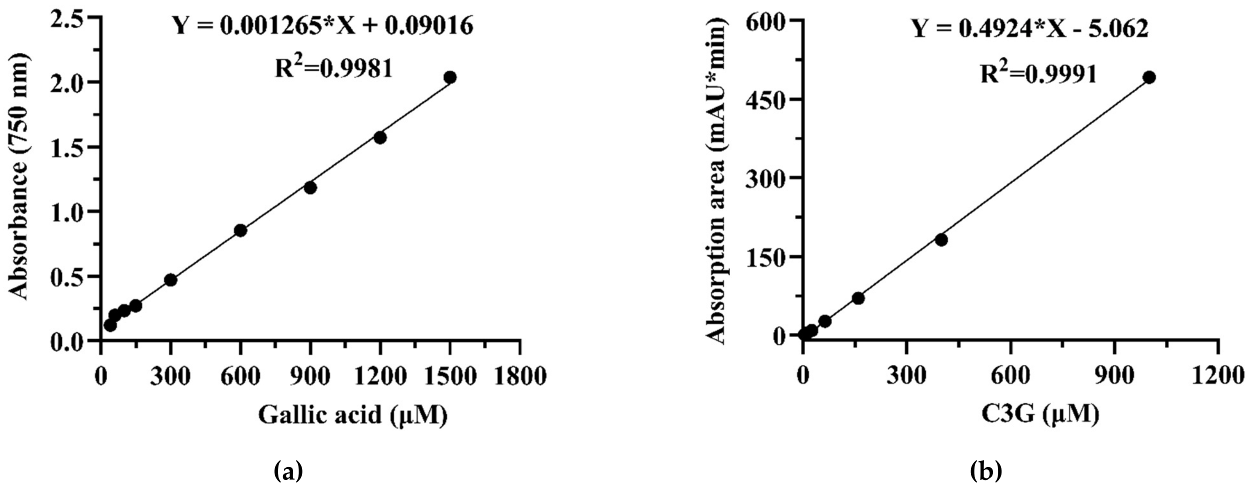

Figure S3. Calibration curve of standard Cyanidin-3-O-glucoside.

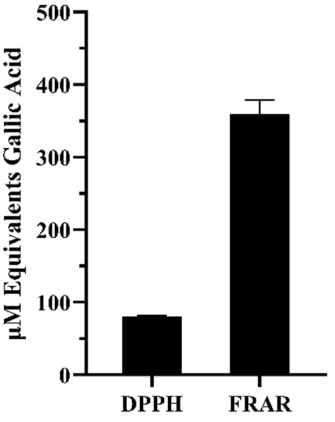

Figure S4. The FRAP and DPPH radical scavenging capacity of the C3G.

Table S1. The primer sequences.

Gene	Forward primer	Reverse primer
GAPDH	GGCAAGTTCAACGGCACAG	CGCCAGTAGACTCCACGACAT
Nrf2	AGTCCAGAAGCCAAACTGACAGAAG	GGAGAGGATGCTGCTGAAGGAATC
NQO1	AAGCCGCAGACCTTGTGATATTCC	CATGGCAGCGTAAGTGTAAGCAAAC
